# Monitoring emerging pathogens using negative nucleic acid test results from endemic pathogens in pig populations: Application to porcine enteric coronaviruses

**DOI:** 10.1371/journal.pone.0306532

**Published:** 2024-07-05

**Authors:** Ana Paula Serafini Poeta Silva, Guilherme Arruda Cezar, Edison Sousa Magalhães, Kinath Rupasinghe, Srijita Chandra, Gustavo S. Silva, Marcelo Almeida, Bret Crim, Eric Burrough, Phillip Gauger, Christopher Siepker, Marta Mainenti, Michael Zeller, Rodger G. Main, Mary Thurn, Paulo Fioravante, Cesar Corzo, Albert Rovira, Hemant Naikare, Rob McGaughey, Franco Matias Ferreyra, Jamie Retallick, Jordan Gebhardt, Angela Pillatzki, Jon Greseth, Darren Kersey, Travis Clement, Jane Christopher-Hennings, Melanie Prarat, Ashley Johnson, Dennis Summers, Craig Bowen, Kenitra Hendrix, Joseph Boyle, Daniel Correia Lima Linhares, Giovani Trevisan

**Affiliations:** 1 Iowa State University, Ames, Iowa, United States of America; 2 University of Minnesota, Saint Paul, Minnesota, United States of America; 3 Kansas State University, Manhattan, Kansas, United States of America; 4 South Dakota State University, Brookings, South Dakota, United States of America; 5 Ohio Animal Disease and Diagnostic Laboratory, Reynoldsburg, Ohio, United States of America; 6 Purdue University, West Lafayette, Indiana, United States of America; National Center of Biotechnology (CNB-CSIC), SPAIN

## Abstract

This study evaluated the use of endemic enteric coronaviruses polymerase chain reaction (PCR)-negative testing results as an alternative approach to detect the emergence of animal health threats with similar clinical diseases presentation. This retrospective study, conducted in the United States, used PCR-negative testing results from porcine samples tested at six veterinary diagnostic laboratories. As a proof of concept, the database was first searched for transmissible gastroenteritis virus (TGEV) negative submissions between January 1^st^, 2010, through April 29^th^, 2013, when the first porcine epidemic diarrhea virus (PEDV) case was diagnosed. Secondly, TGEV- and PEDV-negative submissions were used to detect the porcine delta coronavirus (PDCoV) emergence in 2014. Lastly, encountered best detection algorithms were implemented to prospectively monitor the 2023 enteric coronavirus-negative submissions. Time series (weekly TGEV-negative counts) and Seasonal Autoregressive-Integrated Moving-Average (SARIMA) were used to control for outliers, trends, and seasonality. The SARIMA’s fitted and residuals were then subjected to anomaly detection algorithms (EARS, EWMA, CUSUM, Farrington) to identify alarms, defined as weeks of higher TGEV-negativity than what was predicted by models preceding the PEDV emergence. The best-performing detection algorithms had the lowest false alarms (number of alarms detected during the baseline) and highest time to detect (number of weeks between the first alarm and PEDV emergence). The best-performing detection algorithms were CUSUM, EWMA, and Farrington flexible using SARIMA fitted values, having a lower false alarm rate and identified alarms 4 to 17 weeks before PEDV and PDCoV emergences. No alarms were identified in the 2023 enteric negative testing results. The negative-based monitoring system functioned in the case of PEDV propagating epidemic and in the presence of a concurrent propagating epidemic with the PDCoV emergence. It demonstrated its applicability as an additional tool for diagnostic data monitoring of emergent pathogens having similar clinical disease as the monitored endemic pathogens.

## 1. Introduction

Pathogen monitoring and surveillance systems are routine measures for human and veterinary medicine and recognized as tools for efficient disease control and prevention in populations [1]. Monitoring and surveillance systems’ primary goal is the timely and accurate identification of (re)-emerging pathogens (i.e., minimal or no false alarms). Different types of data can be implemented, e.g., general passive surveillance (based on reports of clinical signs), routine laboratory submissions, animal movement inspections, livestock markets, and other secondary data sources [1]. Dórea et al. [2] identified at least 13 pathogen monitoring systems in animal health while Wendt et al. [3] identified 20 surveillance systems integrating animal and human health.

The ongoing monitoring of animal health parameters and routine monitoring of laboratory submissions test results can reveal trends of pathogen activity, seasonality, and pathogen emergence. In the Netherlands, a national cattle health surveillance system (CHSS) monitors several indicators regarding cattle health, such as mortality, fertility, udder health, and antimicrobial usage [4]. The CHSS detected increases in calf mortality in 2006, which was eventually attributed to the emergence of Bluetongue virus serotype 8 (BTV-8) in 2006 and 2008 [4]. In the United States (US), the Swine Disease Reporting System (SDRS; http://www.fieldepi.org/SDRS) is a surveillance program that was initiated in 2017 and aggregates and standardizes diagnostic test results data from six veterinary diagnostic laboratories (VDLs) with a high swine caseload [5]. The SDRS monitors the detection of nucleic acid by polymerase chain reaction (PCR) or reverse transcription PCR (RT-PCR), using real-time PCR methods, and provides monitoring of nine endemic pathogens of importance to the US swine population: transmissible gastroenteritis virus (TGEV), porcine epidemic diarrhea (PEDV), porcine delta coronavirus (PDCoV), porcine reproductive and respiratory syndrome virus (PRRSV) types 1 and 2 (PRRSV-1 and PRRSV-2), *Mycoplasma hyopneumoniae* (MHP), influenza A virus (IAV), and porcine circovirus type 2 and type 3 (PCV2 and PCV3).

Using data stored in the SDRS, Trevisan et al. [6] monitored the weekly percentage of PCR-positive submissions for PRRSV RNA and identified alarms due to increased PRRSV activity from submissions containing samples from wean-to-market pigs in 2018. In the same study, the surveillance algorithm identified the seasonal cyclic pattern of PRRSV RNA detection with a consistent increase in detection over the years occurring during fall and winter months [6]. Also, an increase in the detection of PRRSV RNA in wean-to-market farms proceeded the increase in detection in sow farms observed at the second half of the year [7]. These studies showed that monitoring and prediction capabilities of surveillance algorithms were signaling early changes from expected detection and pathogen emergence to stakeholders in the cattle and swine industry, who can manage or increase interventions and biosecurity practices to prevent new occurrences in the population.

TGEV is a coronavirus that causes acute enteric disease characterized by diarrhea and vomiting, particularly affecting suckling piglets. Transmissible gastroenteritis virus RNA had been routinely detected by PCR in US VDLs since 2008 [8]. However, at the end of April 2013, US VDLs started receiving submissions of enteric disease that consistently tested negative for TGEV [9]. It was later determined that the enteric disease was associated with the emergence of a transboundary pathogen, PEDV. Like TGEV, PEDV belongs to the family *Coronaviridae*, genus *Alphacoronavirus*, and causes acute diarrhea, vomiting, dehydration, and high mortality in seronegative neonatal piglets [10, 11]. The PEDV emergence caused tremendous economic impact, decreasing the total number of sows farrowing by 0.25%, the number of pigs per litter by 3.0%, and the total number of commercial slaughtered hogs by 4.6% [12].

Even though the PEDV emergence was determined to have occurred in late April and the beginning of May 2013, other PEDV strains, such as the spike gene mutant (S-INDEL) and the S2aa-del strain, emerged in February 2014, subsequently prolonging the PEDV impact in the US swine population [13]. Once PEDV emerged at the end of April 2013, various PCR tests became readily available and testing for TGEV was often concurrently performed and continued to increase substantially [8]. Another coronavirus, PDCoV, was reported to have emerged in the US in February 2014, resulting in a similar but often milder enteric disease as TGEV; however, testing of banked samples revealed PDCoV-positive samples originating from the summer of 2013 [14, 15]. Thus, the continued increase in TGEV diagnostic testing may also have been associated with the PDCoV emergence, suggesting that surveillance systems could also use negative results from monitored endemic pathogens to detect emerging pathogens.

There is no report in the literature of an implemented monitoring system using PCR-negative results in veterinary sciences. Therefore, this study aimed to evaluate different surveillance models that can monitor PCR-negative testing results for endemic enteric viruses and detect changes, which may indicate the potential emergence of a pathogen with similar clinical presentation but lacking routinely used diagnostic assays. To prove the concept, real diagnostic data on TGEV PCR-negative results between 2010 and 2013 were used for a negative result-based monitoring system for enteric coronaviruses during the time of emergence of PEDV in 2013 while TGEV and PEDV PCR-negative results between 2009 and 2014 were used to monitor the PDCoV emergence in 2014. The same methodology was thereafter applied to monitor enteric coronavirus negative results from 2023.

## 2. Materials and methods

### 2.1 Data source

The data used in this study was retrieved from the SDRS (https://www.fieldepi.org/sdrs/), which is an ongoing monitoring project that aggregates producer anonymized diagnostic test results [5, 6, 8] from six participating US VDLs (Iowa State University VDL, University of Minnesota VDL, Kansas State University VDL, South Dakota State University Animal Disease Research and Diagnostic Laboratory, Ohio Animal Disease and Diagnostic Laboratory, and Purdue Animal Disease and Diagnostic Laboratory) and reports the temporal and regional spatial patterns of primary endemic and emerging pathogens in free-of-charge online dashboards and monthly PDF reports. The methodology implemented in the SDRS to collect, process, and standardize diagnostic test results was described elsewhere [5, 6, 8]. Briefly, received producer, veterinarian, farm, and VDL clientele anonymized test result data was standardized across labs. The information used in this study and retrieved from the SDRS database included the unique submission identifier, US State, received date, specimen, pig production phase category (adult/sow farm [breeding herds, replacement, boar stud, suckling piglets, and adults], wean-to-market [nursery and grow-finish], and unknown [no information was provided during submission]), pathogen tested (IAV, MHP, PCV2, PCV3, PEDV, PDCoV, PRRSV-1, PRRSV-2, and TGEV), and PCR testing results (negative, positive, inconclusive, and suspect).

### 2.2 Data management

The observation unit in the study was a porcine diagnostic submission shared with the SDRS database, which was searched for submissions between January 2009, and October 2023. Each submission in the database was assigned a unique identifier. A submission could include PCR testing for one or more pigs, one or more sample types, and one or more pathogens, such as IAV, MHP, PCV2, PCV3, PRRSV-1, PRRSV-2, TGEV, PEDV, or PDCoV. This study focused on PCR testing for enteric coronaviruses (i.e., TGEV, PEDV, and PDCoV).

The PCR results, including negative, suspect, inconclusive, and positive results, were grouped by the accession identifiers, allowing to obtain one submission with related tests in each row. Subsequently, submissions that included only PCR-negative results for TGEV until April 2013 (used to detect PEDV emergence), and then TGEV and PEDV until December 2014 (used to detect PDCoV emergence) were separated into different datasets. That is, submissions that had at least one “suspect”, “inconclusive”, or “positive” result were not accounted for in the analysis of negative submissions. Counts of the total number of negative and positive PCR submissions were calculated on a weekly basis, using the received date at the VDLs as the aggregate factor. This step was repeated for TGEV, PEDV, and PDCoV PCR-negative and positive results.

### 2.3 Data analyses

For this study, an alarm signalized potential danger and deserved further investigation. Thus, a true alarm represented the weeks when TGEV-negative submissions counts were higher than what was predicted by surveillance algorithms, while false alarms represented the alarms detected in the baselines. Therefore, the primary goal of the data analyses in the study was to select an alarm surveillance algorithm that includes one or a combination of surveillance algorithms that were previously validated for diagnostic data monitoring [16]. Additionally, the goal of the selected surveillance algorithms was to maximize early detection while minimizing false alarms to ensure effectiveness when detecting a sustained increased in negative submissions that could indicate the start of an outbreak or epidemic [16, 17]. In this study, a "peak" was defined as an alarm detected in a singular week in the number of TGEV-negative submissions. In contrast, a "sustained increase" refers to a prolonged period, e.g., more than two consecutive weeks of alarms in TGEV-negative submission counts, indicating a potential emerging pathogen. This distinction was necessary as it reduced the likelihood of false alarms by focusing on persistent trends rather than isolated increases. Yet, any type of detected alarm required further investigation. All analyses were done in R (R version 4.2.2, R Core Team 2023, Vienna, Austria, https://www.R-project.org/).

#### 2.3.1 Temporal pattern assessment

Time series were first created to investigate the historical temporal pattern of TGEV PCR-negative (-positive) weekly counts between 2009 and 2014. Weekly counts of submissions with only TGEV PCR-negative results were indexed by the week date (ISO week date standard, ISO-8601). The time series of TGEV PCR-negative submissions were then compared with the time series of positive submissions for PEDV in 2013, while the combination of TGEV and PEDV PCR-negative submissions were compared with PCR-positive submissions for PDCoV in 2014, to determine which weekly time points in TGEV and TGEV and PEDV PCR-negative submissions that were preceding PEDV and PDCoV emergences. Time series were created using *surveillance* and *stats* R packages.

#### 2.3.2 Time series smoothing

Seasonal Autoregressive-Integrated Moving-Average (SARIMA) algorithms were employed to smooth the time series of negative submissions. The purpose of the smoothing process was to control for outliers, abrupt changes, trends, and seasonality to prevent false alarms from being triggered by anomalies that are not indicative of an actual outbreak [18]. For example, an increase in negative submissions following a holiday or as a result of extensive monitoring of negative populations could lead to misleading alarms.

Before performing the SARIMA and given the known changes in historical data of TGEV PCR testing, such as the emergence of PEDV in 2013 and PDCoV in 2014, an iterative outlier detection algorithm proposed by Chen and Liu [19] was implemented to determine specific week points at which the time series exhibited abrupt changes in slope or step-change. The breakpoints were found using the function “tso” from the *tsoutliers* R package. Thereafter, the breakpoints were added as abrupt changes (namely interventions) in the time series to improve their performance [20, 21]. The SARIMA model with intervention, also known as the SARIMA-X model, is an extension of the traditional SARIMA model that incorporates the presence of abrupt changes due to an external event (such as concurrent PEDV outbreak during the PDCoV emergence) in the time series data, accounting for its effects [20]. Different SARIMA models for each period between breakpoints were constructed and their performance evaluated. This approach allowed for a more accurate modeling of the time series and capturing any temporal dynamics associated with the external interventions.

Different time intervals of TGEV-negative data for time series were also evaluated for their ability to select the best-performing SARIMA parameters, e.g., 24 months, 28 months, 30 months, 34 months, and 36 months. These time series sizes were included because SARIMA uses one year as a training set to estimate SARIMA parameters, e.g., (p,d,q)(P,D,Q)s while some detection algorithms (section “*2*.*3*.*3 Anomaly detection algorithms*”) may use several weeks from a whole year to include seasonality in its estimations.

The SARIMA parameters to be estimated are “p” that is auto-regressive (AR) order (the number of lag observations included in the non-seasonal model), “d” integrated (I) order (the number of times that the raw observations are different to make the time series stationary), “q” moving-average (MA) order (the size of the moving average window from the non-seasonal model), “P” seasonal auto-regressive (SAR) order (the number of lag observations included in the -seasonal model), “D” integrated (IS) order (the number of times that the raw seasonal observations are differenced), “Q” seasonal moving-average (SMA) order (the size of the moving average window from the seasonal model), and “s” periodicity (the number of time steps per season; in this study, weeks were used).

The best-performing baseline size of time series and the best performing parameters of SARIMA were selected using the function “auto.arima” from the *forecast* R Package and by visualization of autocorrelation function (ACF) plots. The combination of lowest Root Mean Squared Error (RMSE, representing the average distance between the fitted values and the actual values in the dataset), Mean Absolute Percentage Error (MAPE, representing the average magnitude of error produced by a model), and Akaike Information Criterion (AIC, it was used to select parameters of ARIMA that best fitted the observed data); alongside residual normality (Ljung-Box test p-value < 0.05) were the criteria used to select the best-fitted smoothing procedure [22].

The fitted and residual values from the best-performing SARIMA were then subjected to anomaly detection algorithms to identify any unusual or unexpected anomalies, such as the emergences of PEDV and PDCoV. The SARIMA, SARIMA-X, and pre-selection of parameters for a best-fitted SARIMA were all done using the *forecast* R package.

#### 2.3.3 Anomaly detection algorithms

Different anomaly detection algorithms were tested to assess the performance of the fitted values and residuals estimated from the SARIMA model (Table 1).

**Table 1:** Anomaly detection algorithms tested in the negative-based monitoring system.

Surveillance algorithms	Brief description	Parameters	R package
Cumulative sum control charts (CUSUM)	It is recognized as proper fit for situations where the process average is expected to shift or trend from the specified baseline. CUSUM can use binomial distribution to detect proportional events and Poisson for count events.	**Decision Interval (k)**: it determines the sensitivity, representing the amount of deviation from the target that will trigger an out-of-control signal. **Target Value (h):** the expected value around which the process should operate. **Starting Value (h0):** the starting value of the cumulative sum. It was set to be zero because the process was initially in control. **Directionality (positive or negative):** to detect positive (increase in the process mean) or negative shifts (decrease in the process mean).	*qcc* [23]
Exponentially Weighted Moving Average (EWMA)	It is an exponential smoothing trend and uses the cumulative differences between observed data in a time window and compares it to a threshold, namely, sigma.	**Smoothing Factor (λ):** it determines the weight assigned to the most recent observation, being between 0 and 1. A higher value of λ gives more weight to recent observations, making the EWMA more responsive to changes, while a lower value gives more equal weight to all observations, providing a smoother but less responsive result. **Sigma:** it specifies the number of sigmas to determine upper and lower control limits.	*qcc* [23]
Exponentially Weighted Anomaly Score (EARS)	It is based on the difference between observed values and average values calculated within a moving time window and it standardizes observations using standard deviations from the shorter baselines. It compares the observation against standardized observations from 7 weeks in the past. The EARS includes three methods, namely, “C1” (compares the number of submissions of a week with the average of 7 previous weeks), “C2” (8 weeks), and “C3” (9 weeks).	**Baseline**: number of timepoints from the observation with index baseline + 1 (C1) or + 3 (C2) or + 5 (C3). **Method**: selected between C1, C2, an C3. **Alpha:** approximate prediction interval.	*surveillance* [24]
Farrington and Farrington Flexible	The Farrington algorithm is specifically designed for surveillance data and is commonly used for outbreak detection. It compares observed counts with expected counts at a particular time point but also uses the same time point from previous years using a Poisson distribution. Thus, Farrington algorithms need at least one complete year of baseline to work properly. Noufaily et al. [25] designed a Farrington algorithm, namely Farrington Flexible, to control for false alarms by increasing the re-weight of past weeks.	**Baseline (b)**: number of years back in time to include when forming the base counts. **Window’s half-size**: number of weeks to include before and after the current week in each year. **Weights Threshold**: defines the threshold for reweighting past outbreaks using the Anscombe residuals (1 in the original method, 2.58 advised in the improved method). **Past Weeks Not Included**: number of past weeks to ignore in the calculation. **Alpha**: approximate prediction interval.	*surveillance* [24–26]

Algorithm performance was assessed in terms of false alarm rate and time to detect the outbreak. Time to detect an outbreak refers to the number of weeks between the first alarm and the week reporting emergences of PEDV or PDCoV, while false alarm rate refers to the frequency of incorrect alarms divided by the total number of monitored weeks. For the 2013 PEDV outbreak, the true alarms were expected to begin after April 1^st^, 2013, e.g., one month earlier than the first PEDV disease cases diagnosed in the ISU VDL [9]. This period was chosen to determine true alarms because an epidemiologic investigation identified that PEDV could potentially be circulating a couple of days or weeks before the April 15^th^ index case [11]. Thus, false alarms were defined as any alarms generated in any week between January 2010 and March 29^th^, 2013. For PDCoV, although the first submissions with PCR-positive results occurred in February 2014, there was evidence that the pathogen had been in the US since August 2013 [15]. Therefore, the false alarms were defined as any alarms generated in any week before August 2013.

#### 2.3.4 Application with 2023 detection data

The best performing analytical process(es) (best-performing SARIMA alongside detection algorithms) were then applied to monitor October 2023 data of groups of enteric coronaviruses present and routinely tested in the US [PCR-negative results for all three enteric pathogens, e.g., PEDV, PDCoV, and TGEV].

## 3. Results

### 3.1 PEDV outbreak detection

#### 3.1.1 Temporal pattern assessment

**Fig 1:**
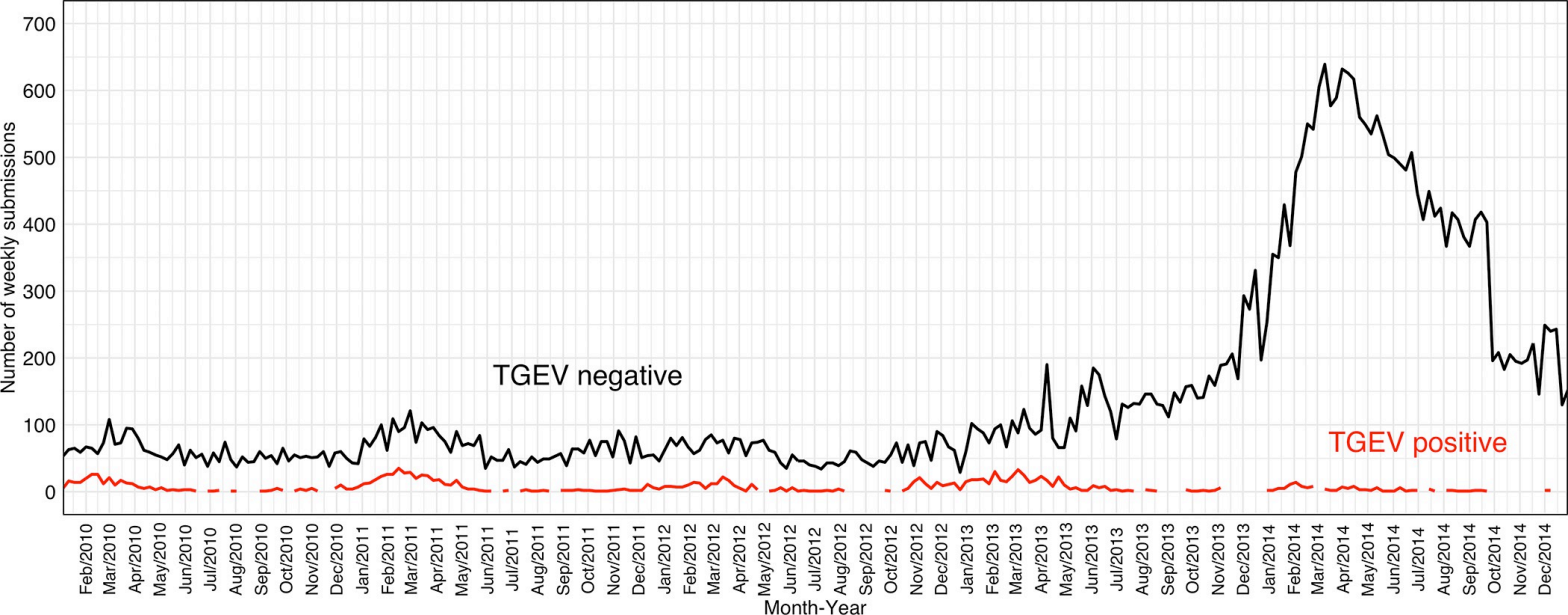
Time-series of transmissible gastroenteritis virus (TGEV) polymerase chain reaction (PCR) results from 2010 through 2014. The black line represents the number of submissions with TGEV PCR-negative results while the red line represents the number of submissions with a TGEV PCR-positive result. The blue rectangle indicates the first peak on TGEV-negative submissions on week of April 8^th^, 2013.

TGEV PCR test results were found in 42,375 submissions recovered between January 5^th^ 2009 and December 29^th^ 2014 from the SDRS database. Of those, 29,429 (70%) were submissions with TGEV PCR-negative results. The time series of TGEV PCR results (negative and positive) from 2010 through 2014 is shown in Fig 1. It was noticeable that TGEV was consistently detected in less than 50 weekly submissions from 2009 through 2014. An increase in TGEV-negative submissions can be observed before the second half of 2013, the noticeable first peak in the raw number of TGEV-negative submissions was on April 8^th^, 2013 (n = 190 negative submissions).

**Fig 2:**
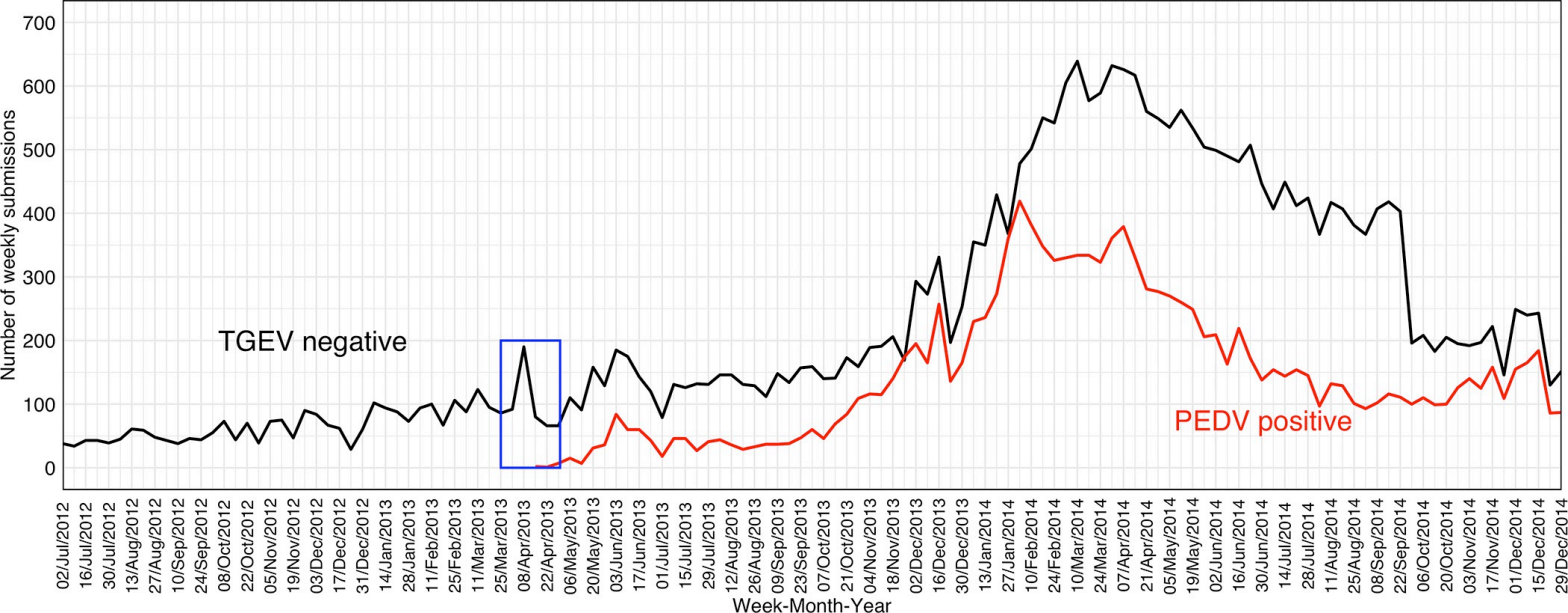
Time-series of transmissible gastroenteritis virus (TGEV) polymerase chain reaction (PCR)-negative results in relation to porcine epidemic diarrhea virus (PEDV) PCR-positive results from 2012 through 2014. The black line represents the number of submissions with TGEV PCR-negative results while the red line represents the number of submissions with at least one PEDV PCR-positive result. The blue square represents the weekly time points (April 8^th^, 2013). in which a potential signal of increased TGEV-negative submissions occurred signalizing the PEDV emergence in the United State in 2013 based on PCR results (April 29^th^, 2013).

Porcine epidemic diarrhea virus PCR test results were found in 54,106 submissions submitted between May 6^th^, 2013, through December 29^th^, 2014. Of those, 37,126 (69%) were PEDV PCR-positive submissions. Fig 2 shows the comparison of TGEV PCR-negative and PEDV PCR-positive submissions. It is noticeable that the increase in TGEV PCR-negative submissions slightly preceded the emergence of PEDV PCR-positive submissions (blue square in Fig 2).

#### 3.1.2 Time series smoothing

**Fig 3:**
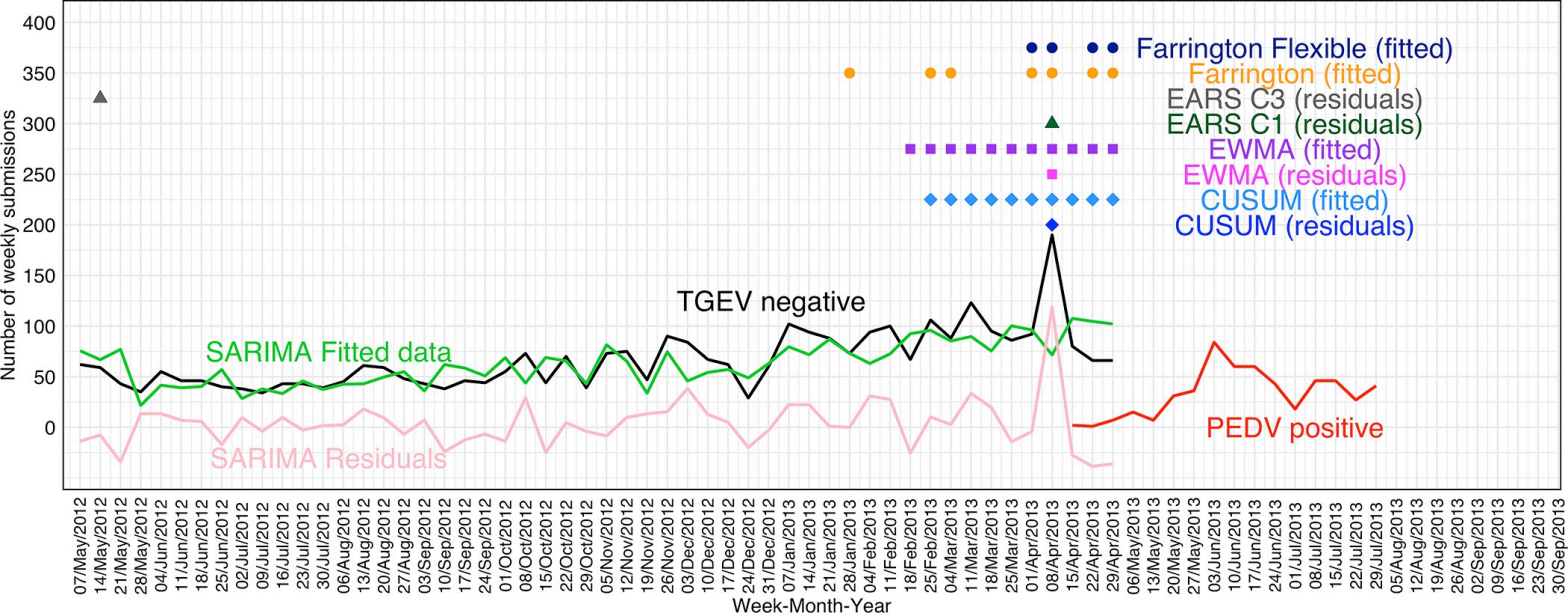
Anomaly detection algorithms (alarms represented by the colored dots) using the transmissible gastroenteritis virus (TGEV) polymerase chain reaction (PCR)-negative submissions (black line) in relation to porcine epidemic diarrhea virus (PEDV) PCR-positive submissions (red line). Seasonal Autoregressive-Integrated Moving-Average (SARIMA) TGEV-negative fitted values (green line), SARIMA TGEV-negative residuals values (pink line).

The best performing smoothing procedure (RMSE = 17.10, MAPE = 12.35%, AIC = 484.74, and Ljung-Box test p = 0.10) was a SARIMA (0,1,1)(0,1,0) with 52 weeks of periodization and included 2 years of data until PEDV outbreak date, e.g., TGEV-negative submissions from May 2^nd^, 2011, through April 29^th^, 2013. The distribution of TGEV-negative submissions used in the SARIMA model, SARIMA TGEV-negative fitted values, SARIMA TGEV-negative residuals values, PEDV-positive submissions are shown in Fig 3. SARIMA model used the first year (May 2^nd^, 2011, through April 29^th^, 2012) as a training set for predictions of the second year (April 30^th^, 2012, through April 29^th^, 2013).

#### 3.1.3 Anomaly detection algorithms

The data from April 30^th^_,_ 2012_,_ through April 29^th^, 2013, was used within the anomaly detection algorithms. That is, the first year of data used by SARIMA model was used as training set, and then it was not used within anomaly detection algorithms. This allowed the tested anomaly detection algorithms to include approximately one year of TGEV negative submissions as baseline, e.g., from April 30^th^, 2012, through March 30^th^, 2013 (approximately 1 month before the PEDV outbreak). As shown in Fig 3 and Table 2, the best-performing anomaly detection algorithm was Farrington Flexible using SARIMA fitted values (false alarm rate 0% and first signal 4 weeks prior to the PEDV outbreak). CUSUM and EWMA using SARIMA fitted values resulted in the earliest detection with consistent alarms but also 5 and 6 false alarms, respectively, e.g., the weeks between February 18^th^ and April 1^st^, 2013. CUSUM, and EARS C1, EWMA using SARIMA residual values resulted in one single alarm, but all on April 8^th^, 2013, where there was a substantial increase in negative submissions.

**Table 2:** Comparison of the performance of detection algorithms following Seasonal Autoregressive-Integrated Moving-Average (SARIMA) smoothing (resulting in fitted and residuals) values using transmissible gastroenteritis virus (TGEV) polymerase chain reaction (PCR)-negative submissions from January 1^st^, 2011, through October 10^th^, 2013. EARS (Exponentially Weighted Anomaly Score), EWMA (Exponentially Weighted Moving Average), and Farrington.

Alarm algorithms	Model parameters	# monitored weeks	Total alarms	False alarms number (%)^a^	Number of true alarms (starting April 1^st^ 2013)^b^	Time (Weeks) to detect PEDV emergence (April 29^th^, 2013)
CUSUM fitted	Initial value of standard error = 0; Number of standard errors to be considered out of control = 5; Number of shifts to be detected = 1	51	10	5 (10%)	5	9
CUSUM residuals	Initial value of standard error = 0; Number of standard errors to be considered out of control = 5; Number of shifts to be detected = 1	51	1	0 (0%)	1	4
EARS C1 fitted	Method = C1; Lambda = 0.5; Alpha = 0.001; Baseline = 7 weeks	51	0	NA	NA	NA
EARS C1 residuals	Method = C1; Lambda = 0.5; Alpha = 0.001; Baseline = 7 weeks	51	1	0 (0%)	1	4
EARS C3 fitted	Method = C3; Lambda = 0.5; Alpha = 0.001; Baseline = 11 weeks	51	0	NA	NA	NA
EARS C3 residuals	Method = C3; Lambda = 0.5; Alpha = 0.001; Baseline = 11 weeks	51	1	1 (2%)	0	NA
EWMA fitted	Baseline = 1 years; Lambda = 0.4; Sigma = 3	51	11	6 (12%)	5	9
EWMA residuals	Baseline = 1 years; Lambda = 0.4; Sigma = 3	51	1	0 (0%)	1	4
Farrington fitted	Baseline = 1 year; Weeks before and after current week = 3 weeks; Past week weight = 1; Number of past weeks not included = 3; Alpha = 0.05	51	7	3 (6%)	4	9
Farrington Flexible fitted	Baseline = 1 year; Weeks before and after current week = 1 weeks; Weight of past week = 2.58; Number of past weeks not included = 3; Alpha = 0.05	51	4	0 (0%)	4	4

^a^ False alarm rate referred to the frequency of incorrect alarms (false positive) divided by the total number of monitored weeks. For PEDV emergence, given that the true alarms were expected to begin after April 1^st^, 2013 (one month earlier than the first PEDV disease cases observed in the ISU VDL, on April 29^th^ 2013), any alarms generated in any weeks between January 2010 and March 29^th^, 2013 were considered false alarms.

^b^ True alarms referred to alarms generated when there was a true increase in weekly negative submissions within the period of monitoring. For PEDV emergence, the true alarms were expected to begin after April 1^st^, 2013 (one month earlier than the first PEDV disease cases observed in the ISU VDL, on April 29^th^, 2013).

^c^ Time to detect an outbreak refers to the number of weeks between the first alarm and the week reporting PEDV emergence (April 29^th^, 2013).

### 3.2 PDCoV outbreak detection

#### 3.2.1 Temporal pattern assessment

**Fig 4:**
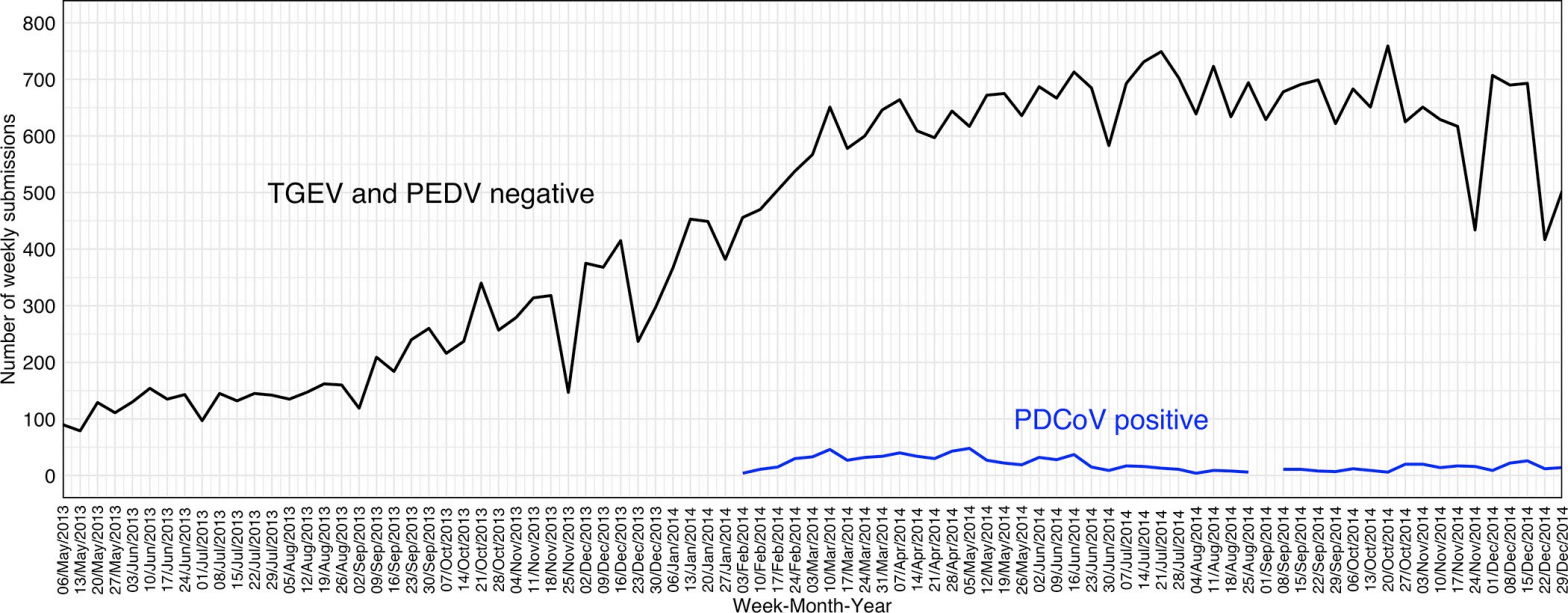
Time-series of transmissible gastroenteritis virus (TGEV) and porcine epidemic diarrhea virus (PEDV) polymerase chain reaction (PCR)-negative in relation to delta coronavirus (PDCoV) PCR-positive results from 2013 through 2014. The black line represents the number of submissions that included TGEV and PEDV PCR-negative results, the blue line represents the number of submissions that included at least one PDCoV PCR-positive result.

Porcine delta coronavirus PCR test results were found in 15,291 submissions from December 12^th^, 2013, through December 29^th^, 2014. Of those, 937 (6%) were PDCoV PCR-positive submissions. The time series of TGEV and PEDV PCR-negative results included a total of 13,020 submissions from January 1^st^, 2012, and December 29^th^, 2014. Of those, 3,799 (29%) were submissions with PEDV PCR-negative results only, 4,557 (35%) TGEV PCR-negative results only, and 4,664 (36%) included both PEDV and TGEV PCR-negative results. The time series between May 2013 (following PEDV emergence) through December 2014 is shown in Fig 4. The highest peak of TGEV and PEDV PCR-negative submissions was aligned with the first peak of PDCoV PCR-positive submissions, i.e., 50 PDCoV-positive submissions on March 10^th^, 2014 (Fig 4). The emergence of PDCoV was milder compared to PEDV, with less than 50 weekly PDCoV-positive submissions.

#### 3.2.2 Time series smoothing

Due to the implementation of PEDV PCRs after its emergence in April 2013, PEDV PCR-negatives were used alongside TGEV PCR-negative result to monitor enteric viruses’ emergences. Therefore, it required that the baseline model was re-set to account for the new trend and seasonality to adjust performance prediction of SARIMA (update parameters). The outlier detection algorithm identified that TGEV PCR-negative submissions had two step changes on June 1^st^, 2013, and October 14^th^, 2013, dates that corresponded to little over a month following PEDV emergence and increasing in PEDV testing, i.e., increase in PEDV PCR-negative and positive submissions (Figs 2 and 4). Thus, the PEDV outbreak was added as an intervention (using a SARIMA-X model) and PEDV PCR-negative submissions were subsequently added to the baseline to monitor the PDCoV emergence in 2013 and 2014, resulting in increase in the SARIMA forecast performance. The best performing smoothing (RMSE = 36.5, MAPE = 11.3%, AIC = 569.3, and Ljung-Box test p = 0.56) was a 2-step SARIMA-X (3,1,0)(0,1,0) with 52 weeks of periodization and included 2 years of data until PDCoV outbreak date, e.g., TGEV- and PEDV-negative submissions from February 6^th^, 2012 through February 3^rd^, 2014 (Fig 4).

#### 3.2.3 Anomaly detection algorithms

**Fig 5:**
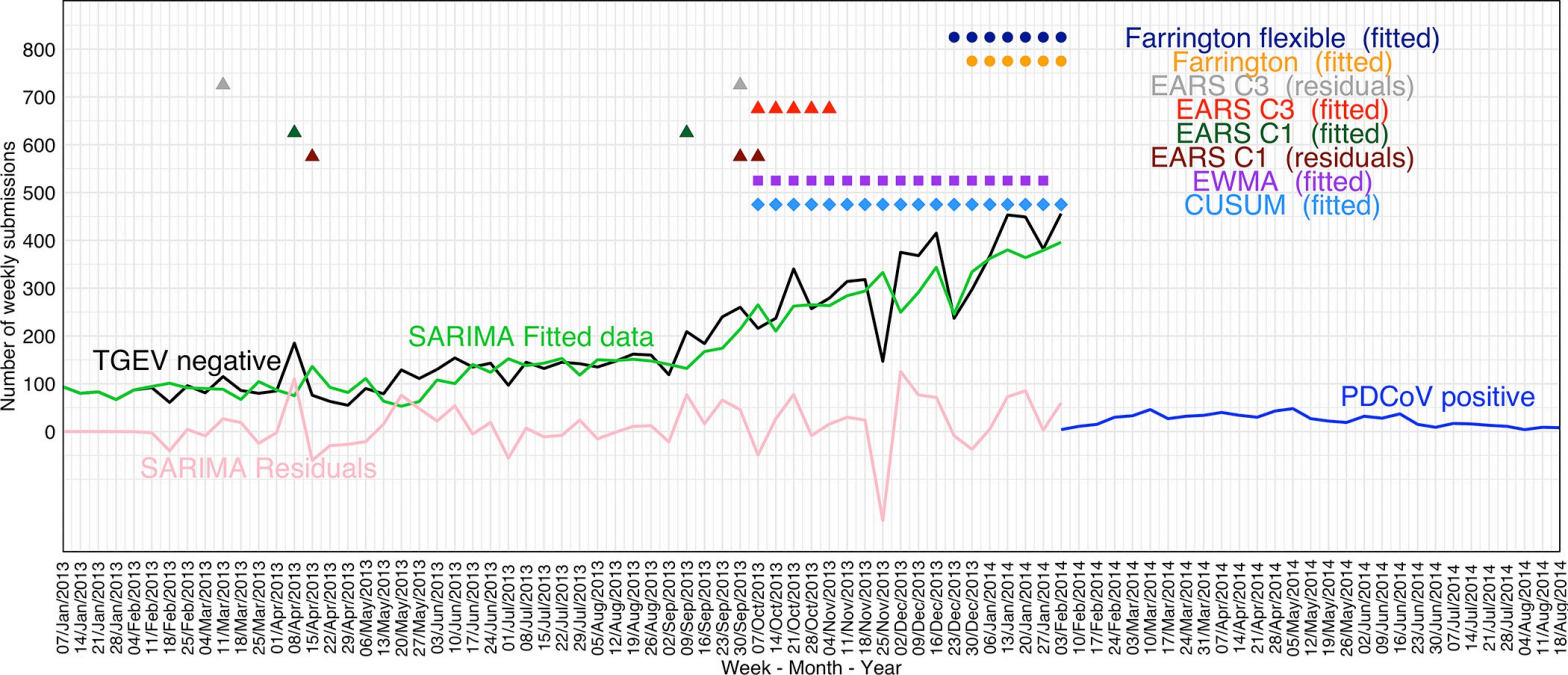
Anomaly detection algorithms (alarms represented by the colored dots) using the transmissible gastroenteritis virus (TGEV) and porcine epidemic diarrhea virus (PEDV) polymerase chain reaction (PCR)-negative submissions (black line) in relation to porcine delta coronavirus (PDCoV) PCR-positive submissions (blue line). Seasonal Autoregressive-Integrated Moving-Average (SARIMA) fitted values (green line), and SARIMA residuals values (pink line).

The data used within the anomaly detection algorithms were from January 7^th^_,_ 2012, through January 31^st^, 2014, and then included 1 year as baseline to monitor TGEV- and PEDV-negative submissions from January 2014 (January 1^st^ to January 31^st^, 2014). The best performing anomaly detection algorithms were Farrington Flexible, with 0% false alarm rate and 4 weeks as the time to detect PDCoV emergences. CUSUM, and EWMA using SARIMA fitted values resulted in earliest detection and consistent detection (Fig 5 and Table 3), no false alarms and time to detect between 12 to 13 weeks before PDCoV emergence. CUSUM and EWMA using SARIMA residual values resulted in one single alarm on January 20^th^, 2014, where there was a substantial increase in negative submissions.

**Table 3:** Comparison of the performance of detection algorithms following Seasonal Autoregressive-Integrated Moving-Average (SARIMA) smoothing (resulting in fitted and residuals) using gastroenteritis virus (TGEV) and porcine epidemic diarrhea virus (PEDV) polymerase chain reaction (PCR)-negative submissions from January 1^st^, 2012, through February 1^st^, 2014, to monitor porcine delta coronavirus (PDCoV) PCR-positive submissions.

Alarm algorithms	Model parameters	# monitored weeks	Total alarms	False alarms (%)^a^	Number of true alarms (starting at June 1^st^ 2013)^b^	Time (Weeks) to detect PDCoV emergence (February 1^st^, 2014)^c^
CUSUM fitted	Initial value of standard error = 0; Number of standard errors to be considered out of control = 5; Number of shifts to be detected = 1	51	17	0 (0%)	17	17
CUSUM residuals	Initial value of standard error = 0; Number of standard errors to be considered out of control = 5; Number of shifts to be detected = 1	51	0	NA	NA	NA
EARS C1 fitted	Method = C1; Lambda = 0.5; Alpha = 0.001; Baseline = 7 weeks	51	1	1 (50%)	1	18
EARS C1 residuals	Method = C1; Lambda = 0.5; Alpha = 0.001; Baseline = 7 weeks	51	3	1 (33%)	2	18
EARS C3 fitted	Method = C3; Lambda = 0.5; Alpha = 0.001; Baseline = 11 weeks	51	5	0 (0%)	5	17
EARS C3 residuals	Method = C3; Lambda = 0.5; Alpha = 0.001; Baseline = 11 weeks	51	1	1 (100%)	NA	NA
EWMA fitted	Baseline = 1 years; Lambda = 0.4; Sigma = 3	51	17	0 (0%)	17	17
EWMA residuals	Baseline = 1 years; Lambda = 0.4; Sigma = 3	51	0	NA	NA	NA
Farrington fitted	Baseline = 1 year; Weeks before and after current week = 3 weeks; Past week weight = 1; Number of past weeks not included = 3; Alpha = 0.05	51	5	0 (0%)	5	5
Farrington Flexible fitted	Baseline = 1 year; Weeks before and after current week = 1 weeks; Weight of past week = 2.58; Number of past weeks not included = 3; Alpha = 0.05	51	6	0 (0%)	6	6

^a^ False alarm rate referred to the frequency of incorrect alarms (false positive) divided by the total number of monitored weeks. For PDCoV emergence, given that the true alarms were expected to begin after June 1^st^, 2013 (8 months earlier than the first PDCoV disease cases on February 1^st^, 2014), any alarms generated in any weeks between January 2012 and June 2013 were considered false alarms.

^b^ True alarms referred to alarms generated when there was a true increase in weekly negative submissions within the period of monitoring. For PDCoV emergence, the true alarms were expected to begin after June 1^st^.

^c^ Time to detect an outbreak refers to the number of weeks between the first alarm and the week reporting PDCoV emergence (February 1^st^, 2014).

Based on performance results in detecting PEDV and PDCoV emergences, a combination of detection algorithms was selected, e.g., Farrington Flexible using SARIMA fitted values, CUSUM and EWMA using SARIMA fitted and residuals, to monitor 2023 negative enteric data.

### 3.3 Application– 2023 negative case data

#### 3.3.1 Enteric-negative submissions

The data used for monitoring enteric-negative submissions were the submissions with PCR-negative results for TGEV, PEDV, and PDCoV between October 24^th^, 2021, and October 24^th^, 2023. Thus, the data included a total of 79,938 submissions with PCR-negative results for the three enteric viruses, whereas 93% (74,399 of 79,938) had PCR-negative results for all three enteric viruses (TGEV, PEDV, and PDCoV), 4% PEDV PCR-negative only, and 3% PEDV- and PDCoV-negative.

**Fig 6:**
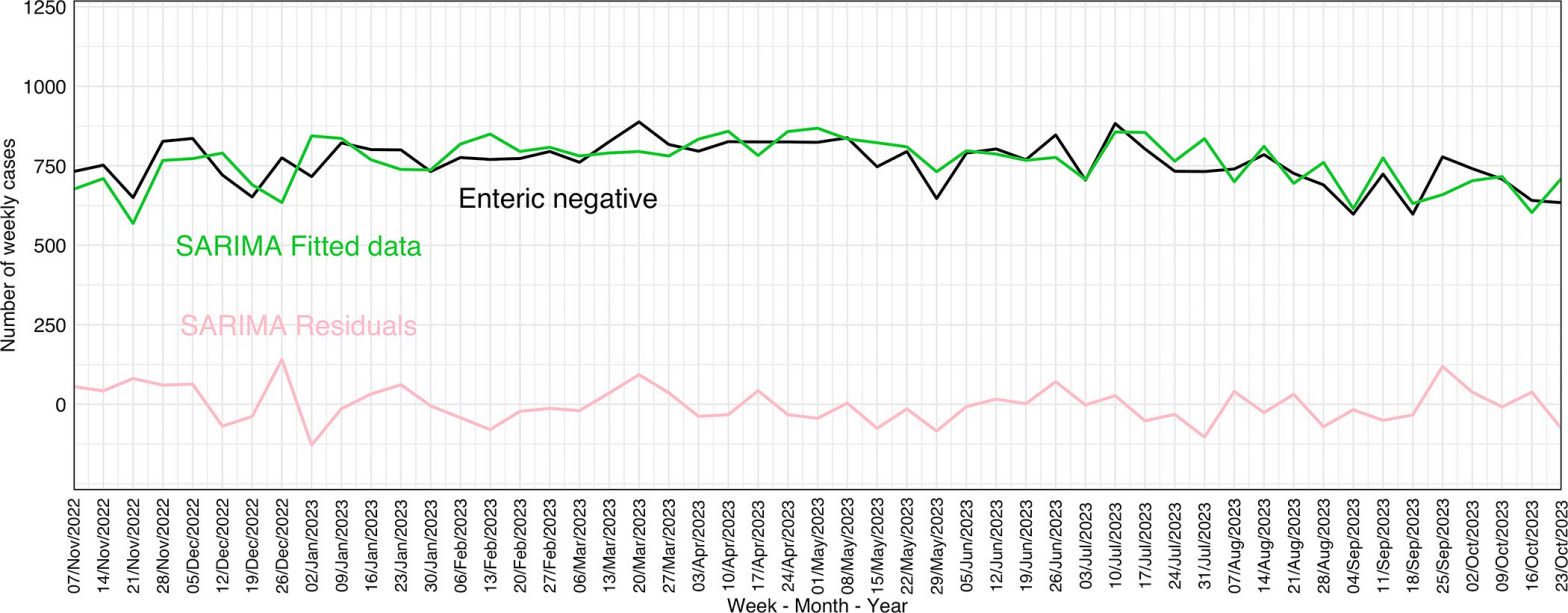
Distribution of enteric polymerase chain reaction (PCR)-negative submissions (black line) in 2023, Seasonal Autoregressive-Integrated Moving-Average (SARIMA) enteric-negative fitted (green line), and SARIMA enteric-negative residuals (pink line).

The best performing smoothing was a SARIMA (5,1,1)(0,1,0) with 52 weeks of periodization (RMSE = 39.5, MAPE = 3.05%, AIC = 582.1, and Ljung-Box test p = 0.18). The negative enteric data used within the anomaly detection algorithms were from October 24^th^, 2022, and October 24^th^, 2023. No alarms were reported with CUSUM and EWMA using fitted and residuals SARIMA values (Fig 6). In contrast, Farrington Flexible using SARIMA fitted values identified two alarms in enteric negative submissions on January 2^nd^ and February 13^th^, 2023. These alarms were considered false alarms because CUSUM and EWMA using fitted SARIMA values did not classify these dates as alarms. Thus, there were no alarms for 2023 negative enteric data (Fig 6).

## 4. Discussion

An outbreak of an emerging pathogen associated with enteric coronavirus-like clinical signs with no routine diagnostic test will likely result in a sustained increase of PCR-negative submissions for other endemic enteric coronavirus. CUSUM and EWMA are statistical control charts that are appropriate to detect sustained increases or decreases and then were appropriate detection algorithms to be used in the negative result monitoring, while controlling for seasonality and trends [18]. While Farrington Flexible performs one-timepoint detection, but also considers seasonality and time trends [25, 26]. Here all three best-performing algorithms CUSUM, EWMA, and Farrington flexible resulted in alarms four weeks earlier than the first disease diagnosis of PEDV in the US. These alarms were considered early true alarms of the PEDV emergence given that an epidemiologic investigation described the first reports of PEDV infections in the US occurred on April 15^th^, 2013 (detection in enteric samples by PCR), and that the virus might have been circulating in the US a few weeks earlier [11]. Ultimately, PEDV-like disease cases started to be diagnosed at US VDLs on April 29^th^, 2013. These results showed that the use of negative monitoring accurately identified the sustained increase in TGEV negative submissions aligned with the emergence of PEDV in 2013.

Given that anomaly detection algorithms are expected to not perform optimally across all scenarios [16], the interpretation of true alarms was done by simultaneous detection by the three detection algorithms. Thus, discordant results across detection algorithms (false alarms) were investigated whether there was an alarm when there was no increase in the observed data or if there were alarms detected due to a sporadic increase (peaks) in observed data in specific weeks. For instance, Farrington flexible identified two alarms in nonconsecutive weeks in 2023 enteric negative submissions, but CUSUM and EWMA detected no sustained increase in the same data that could reveal the emergence of a novel pathogen, and then, those two detections were classified as false alarms. This corresponded to field perspectives and SDRS assessment of patterns with enteric coronaviruses in 2023, e.g., there was no report of an emerging enteric pathogen in 2023. Therefore, the use of three algorithms contributed to distinguish between peak alarms and sustained increase.

MAPE is a scale-independent measure that represents the average magnitude of error considering fitted and raw data values used as criteria to select forecasting models when using time series with different datasets [22]. In the monitoring with TGEV-negative submissions only (by April 29^th^, 2013), 12.34% MAPE was observed. In contrast, MAPEs derived from SARIMA using data from 2023 enteric coronaviruses negative submissions resulted in less than 3%, e.g., a dataset that did not include known outbreaks. These results showed that the emergence of pathogens will likely alter the SARIMA forecast accuracy and that the model will need to be updated as new external events occur.

Before the PEDV emergence in 2013 (Fig 1), the seasonality of TGEV-negative submissions included a similar pattern as TGEV-positive submissions, in which the increase in TGEV-negative submissions was aligned with the increase in TGEV-positive submissions during fall and winter seasons [8]. Thereafter, the pattern of weekly TGEV PCR-negative submissions visually followed the pattern of PEDV detection between May 2013 through December 2014. Still, there were various peaks of increased submissions starting mid-February 2013, e.g., almost three months before the PEDV emergence. This was signaled by two detection algorithms, CUSUM and EWMA, but not by Farrington flexible. Alarms between mid-February and mid-March 2013 may be due to the increase in TGEV submissions, as reported elsewhere [8]. Likewise, only Farrington Flexible resulted in alarms consistent with enteric coronavirus-negative submissions during January and February 2023. These alarms may also be associated with increased PCR submissions for enteric coronaviruses during the winter season. These results suggested that the pattern of PCR submissions may influence the sporadic alarms detected in negative results, and then, the negative monitoring will work as an ally with the monitoring of PCR-positive endemic pathogens.

PDCoV emergence was observed as an emergence of lower magnitude compared to PEDV. Still, PDCoV circulation also increased the pattern of TGEV and PEDV PCR-negative results alongside the PEDV emergence, as shown by the sustained alarms provided by CUSUM, EWMA, and Farrington. Continued monitoring during an ongoing outbreak following herd’s negative outcomes of routine testing can support the identification of an emerging pathogen. Despite its emergent status in the US since August 2013, PDCoV detection remained low in 2014. However, the detection algorithms suggested that PDCoV alarms were missed over three months. This could be explained by the clinical presentation of PDCoV, as TGEV, PDCoV is recognized to be associated with a less severe enteric disease when compared to PEDV [27, 28], and because PDCoV emerged short after the PEDV emergence, its initial detection may have been neglected. Additionally, health interventions and biosecurity measures taken against PEDV in the summer of 2013 likely decreased TGEV occurrences and ameliorated PDCoV impact in 2013 and 2014, resulting in lower detection of PDCoV in following years.

In a recent example of an emergent pathogen, a swine enteric disease outbreak was observed in pig farms in Guangdong province, China, in October 2016 [29]. The farms were endemic for PEDV (PEDV PCR-positive in enteric piglet tissue), with diagnostic testing for PEDV or other enteric viruses consistently pursued. However, after January 2017, negative results for PEDV and other enteric pathogens were observed, despite the rising mortality and enteric clinical signs in sows and piglets. A novel virus was eventually identified and described as the swine acute diarrhea syndrome coronavirus (SADS-CoV), which includes bats as reservoirs and can infect pigs and humans [30]. SADS-CoV has re-emerged in other provinces of China in 2021, highlighting its spread in the country [31]. SADS-CoV has not been reported in the US and, therefore, is not part of routine diagnostic testing in US VDLs. Upon emergence of a novel enteric coronavirus in the US, such as SADS-CoV, diagnosis of TGEV, PEDV, and PDCoV would be first targeted by practitioners and diagnosticians. The emergence of swine enteric coronaviruses in other regions highlights the need to continually monitor negative results from endemic enteric coronaviruses in the to enhance the monitoring capability for emerging enteric pathogens.

Ongoing data collection and curation are essential steps to implement in diagnostic data monitoring systems [16]. This study was limited to the monitoring of negative enteric coronaviruses PCR testing results, and thus, the performance of the proposed monitoring system will increase as more data become aggregated and standardized for other endemic enteric pathogens, such as rotaviruses and *Escherichia coli* [32].

Still, caution is required when interpreting alarms on these data. As discussed elsewhere [8], this study used PCR test results (other virologic diagnostic test were not accounted in the monitoring) that represented conclusions based on samples submitted for diagnostic testing and did not represent disease occurrence, prevalence, or incidence. Additionally, the number and frequency of samples are also driven by submission purpose (i.e., surveillance and monitoring, diagnosis of disease, creation of tissue-homogenate inoculum), the economics of a particular disease, and availability and the cost of diagnostic testing. Additionally, this study used realized PCR-detection data with known outbreak events, i.e., PEDV and PDCoV emergence in the US, for algorithm development and validation. No simulation and injection of peaks or alarms was performed on the data to keep its natural nature of field occurrence and diagnostic testing to evaluate how well the monitoring algorithms would perform.

The performance of SARIMA and anomaly detection algorithms is attached to the selection of proper baselines. Therefore, external events and variations on the baseline will demand periodic revision of model parameters. The SDRS utilizes an advisory group including field veterinarians and stakeholders in VDLs, industry, and academia who are continuously contacted to determine the plausibility of alarms (true and false alarms) and use the outcomes for making evidence-based, clinically relevant decisions regarding pathogen detection, control and prevention [8].

Early detection of novel pathogen emergence, even without immediate identification of the specific pathogen, will provide stakeholders with opportunities for proactive responses (enhanced biosecurity protocols and surveillance), biocontainment (contain the spread of the pathogen), resource allocation (increased surveillance in pathogen-free pig populations), diagnostics (accelerated diagnostic developments), awareness (raising awareness among producers and veterinarians can lead to early reporting of unusual symptoms and faster pathogen identification).

## 5. Conclusions

This study proposed a monitoring system using negative results from enteric coronavirus PCR testing in the US, in which the primary goal is the early identification of a sustained increase in negative submissions that could indicate that a novel pathogen has emerged. To prove the concept, real diagnostic testing data on TGEV PCR-negative results preceding the rise in detection of PEDV infections in 2013 and real diagnostic testing data on TGEV and PEDV PCR-negative preceding PDCoV in 2014 were used. These steps revealed the best-fitted smoothing SARIMA model and a combination of best-performing detection algorithms to be applied to prospective negative enteric data. The retrospective analysis showed that the negative-based monitoring system will function in case of a propagating epidemic (like PEDV emergence in the US) and a secondary emerging pathogen (PDCoV) in the presence of a concurrent propagating epidemic. External events and variations on the baseline will demand periodical revision of model parameters for monitoring prospective negative submissions. The prospective PCR-negative-based monitoring system used time series, including 2 years of PCR-negative results from TGEV, PEDV, and PDCoV, and SARIMA to control for seasonality, outliers, and abrupt changes. The SARIMA’s fitted and residual values were subjected to the three best-performing anomaly detection algorithms (CUSUM, EWMA, and Farrington flexible), which provided earliest detection and minimal false alarms. The monitoring system revealed no alarms for 2023 negative PCR enteric data.
